# C–H bond functionalization: recent discoveries and future directions

**DOI:** 10.3762/bjoc.19.114

**Published:** 2023-10-17

**Authors:** Indranil Chatterjee

**Affiliations:** 1 Department of Chemistry, Indian Institute of Technology Ropar, Nangal Road, Rupnagar, Punjab 140001, India

**Keywords:** C–H bond functionalization

The process of C–H bond functionalization can be defined as the replacement of an activated or nonactivated C−H bond with a functional group. This discipline surfaced within the last few decades and proved to be a powerful synthetic tactic due to some remarkable advantages. It has drawn immense attention from the scientific community, thanks to some significant opportunities, such as the use of readily available feedstocks, the introduction of functionality at specific positions of molecules without requiring any prefunctionalized precursors, and the conversion of light alkanes to higher-value analogues [1–2]. The nonnecessity of prefunctionalization provides a step-economic alternative to classical reactions as well as famous Noble-prize-winning cross-couplings, therefore approaching another step up towards sustainability.

Likewise, a free-radical process is also a classical way to functionalize nonactivated C−H bonds in which site selectivity arises either from the relative strength of the C−H bonds or from the abstraction of intramolecular hydrogen atoms. Radical chemistry is a viable alternative to the two-electron process, involving C–H bond functionalization in the absence of any ligand and using low-cost redox-active metals (Fe, Cu, Mn, etc.) rather than heavy metals (Rh, Ir, etc.). Although radical strategies are age-old processes, they were initially cumbersome due to the stoichiometric use of heavy metal salts, peroxides, and other toxic materials as well as the generation of heavy organic and inorganic wastes. In modern days, new strategies are being developed, dealing with photoredox chemistry and its combination with organometallic chemistry for site-selective C−H bond functionalization [3–4]. Recent years have witnessed many viable strategies for the synthesis of complex targets utilizing photoredox catalysis, electroorganic catalysis, Lewis acid catalysis, and transition-metal-free techniques. Some energy-economic reactors such as ball mill, microwave, ultrasound and, most importantly, flow reactors have also evolved towards a more sustainable future.

To showcase the modern approaches in this domain, this thematic issue in the *Beilstein Journal of Organic Chemistry* gathers recent reports from several research groups, including photochemical as well as transition-metal-mediated C–H bond functionalization. This mixing of traditional and classical with modern-day research will surely encourage synthetic chemists to sketch new methodologies.

Indranil Chatterjee

Rupnagar, September 2023
